# From acute stress syndrome to CNS infection: A case of HSV encephalitis with psychiatric mimicry

**DOI:** 10.1097/MD.0000000000046546

**Published:** 2025-12-12

**Authors:** Riazullah, Aafeen Mujeeb, Muhammad Saqib, Inibehe Ime Okon

**Affiliations:** a Department of Medicine, Mardan Medical Complex, Mardan, Pakistan; b Department of Medicine, Khyber Medical College, Peshawar, Pakistan; c Department of Research, Medical Research Circle (MedReC), Bukavu, DR Congo.

**Keywords:** acute stress syndrome, case report, cns infection, encephalitis, HSV

## Abstract

**Rationale:**

Herpes simplex virus type 1 (HSV-1) encephalitis is a rare but life-threatening central nervous system infection. Its early manifestations can mimic primary psychiatric disorders, which may delay recognition and treatment. We report a case where acute stress disorder masked underlying HSV-1 encephalitis.

**Patient concerns:**

A 47-year-old male patient presented with acute emotional distress, anxiety, sleep disturbance, and dissociative symptoms following the sudden death of his young niece.

**Diagnoses:**

The patient initially fulfilled Diagnostic and Statistical Manual of Mental Disorders, Fifth Edition criteria for acute stress disorder without neurological signs. During hospitalization, he developed fever, confusion, and disorientation. A computed tomography scan was initially interpreted as normal; however, subtle temporal lobe hypodensities were noted by a medical trainee. A subsequent magnetic resonance imaging revealed temporal lobe hyperintensities. cerebrospinal fluid analysis showed lymphocytic pleocytosis, and polymerase chain reaction confirmed HSV-1, establishing the diagnosis of HSV-1 encephalitis.

**Interventions:**

The patient was treated with intravenous acyclovir.

**Outcomes:**

The patient’s fever and psychiatric symptoms improved, and he was discharged in stable condition after completing a 7-day course of antiviral therapy. At a 1-month follow-up, he showed no neurological or psychiatric sequelae.

**Lessons:**

There are diagnostic challenges when psychiatric symptoms occur before neurological manifestations of HSV encephalitis. Clinicians should maintain a high index of suspicion for organic etiologies in atypical psychiatric presentations, and early neuroimaging and cerebrospinal fluid studies are essential to avoid delays in life-saving treatment.

## 1. Introduction

Encephalitis is an acute infection of the brain parenchyma characterized by fever, headache, and altered consciousness. Neurologic deficits and seizures may also occur.^[1]^ Among viral causes, herpes simplex virus (HSV) is the most common etiology, manifesting as life-threatening herpes simplex encephalitis or benign aseptic meningitis.^[2]^

Reactivation of latent HSV is associated with immunosuppression or stressors, but reports linking acute psychological stress, particularly acute stress disorder (ASD), with HSV reactivation remain rare. ASD describes post-traumatic stress reactions within 1 month of trauma, with diagnostic criteria including dissociative, intrusive, avoidant, and arousal symptoms.^[3,4]^

Previous literature has documented psychiatric mimicry of HSV encephalitis, but this case is unique in highlighting how an initial Diagnostic and Statistical Manual of Mental Disorders, Fifth Edition (DSM-5)—consistent diagnosis of ASD delayed recognition of HSV-1 encephalitis and how early vigilance by a medical trainee prevented misdiagnosis.

While acyclovir therapy remains the cornerstone of treatment, delayed initiation increases mortality up to 70%, with fewer than 3% returning to baseline neurologic function.^[5]^ Thus, early diagnosis is essential.

## 2. Case presentation

A 47-year-old male patient presented with severe anxiety, sleep disturbance, emotional distress, and dysregulation following the death of his young niece in a burn accident. He fulfilled DSM-5 diagnostic criteria for ASD, including:

- Intrusive distressing memories of the event;
- Dissociative symptoms (emotional numbing, detachment);
- Avoidant behaviors; and
- The patient exhibited marked arousal symptoms, such as sleep disturbance and irritability.

He was evaluated psychiatrically and started on anxiolytics. No neurologic deficits were present initially, supporting a psychiatric diagnosis.

**Figure 1.**
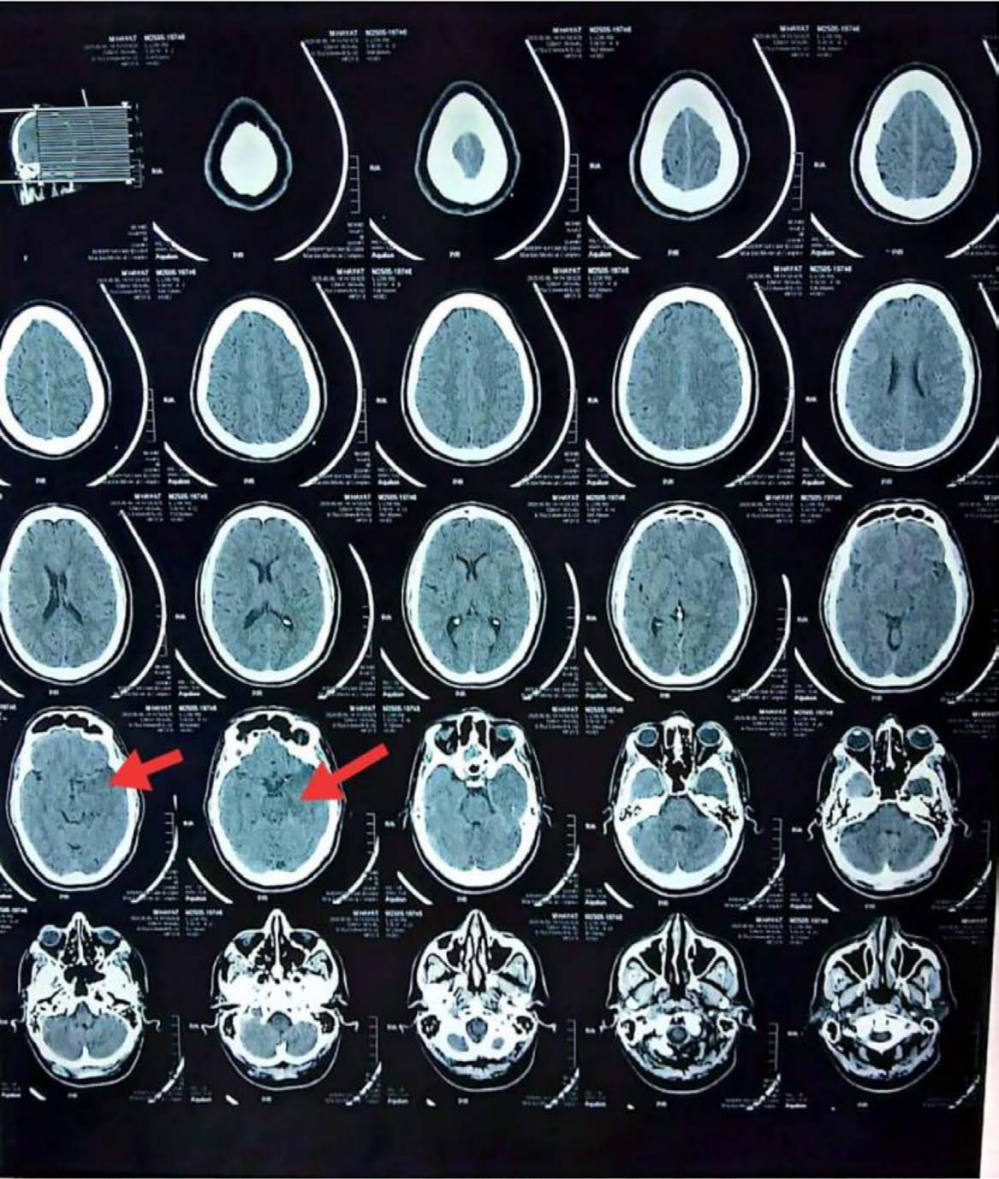
Non-contrast CT brain.

During hospitalization, the patient developed a high-grade fever, irritability, progressive confusion, and disorientation. A non-contrast computed tomography (CT) brain was reported as “normal” by the radiologist. However, a medical trainee observed subtle hypodense lesions in 2 consecutive temporal lobe cuts (Fig. 1) and raised concern for early HSV encephalitis.

**Figure 2.**
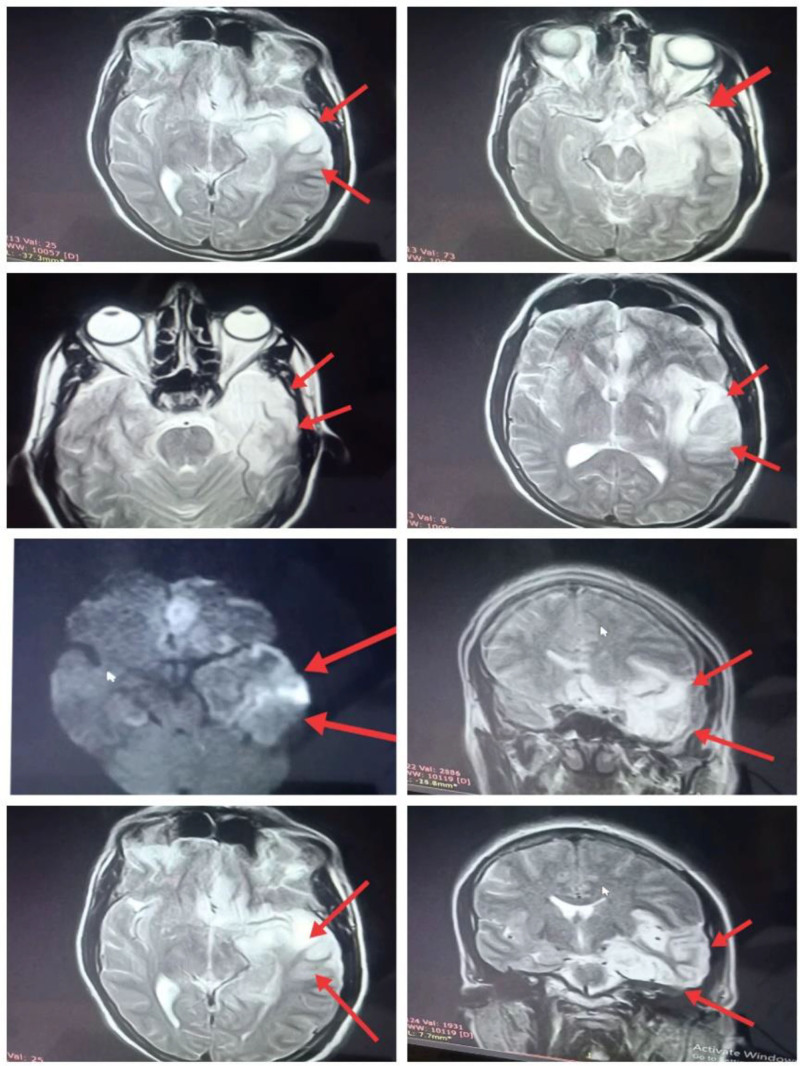
Axial, coronal, and sagittal magnetic resonance brain images on T2-weighted and FLAIR sequences showing hyperintense lesions in the temporal lobe, predominantly involving the medial region.

When asked, the supervising physician initially considered discharge after the fever subsided, but the trainee’s insistence led to an MRI, which revealed temporal lobe hyperintensities highly suggestive of HSV encephalitis (Fig. 2).

Lumbar puncture revealed elevated protein, normal glucose, and lymphocytic pleocytosis (Table 1). Polymerase chain reaction confirmed HSV-1, establishing the diagnosis.

**Table 1:** Cerebrospinal fluid routine examination.

Physical examination	Result	Reference values
Cerebrospinal fluid routine examination		
Volume	02 mL	1.5–2.5 mL
Appearance	Clear	
Deposit	Nil	
Protein	500 mg/dL	20–40 mg/dL
Sugar	30 mg/dL	60–80 mg/dL
LDH	156 U/L	125–220 U/L
Total WBC count	600/cm^3^	0–8/cm^3^
Total RBC count	Numerous	0–1 RBC/cm^3^
Neutrophil	10%	35%–45%
Lymphocytes	90%	40%–70%
ZN stain	Not seen	
Gram stain	No microorganism seen	

LDH = lactate dehydrogenase, WBC = white blood cells, RBC = red blood cells.

The patient was treated with intravenous acyclovir for 14 days (extended beyond 7 due to initial severity). His fever and confusion resolved, and psychiatric symptoms improved. At a 1-month follow-up, he showed no neurologic deficits or psychiatric sequelae. The overall clinical timeline is summarized, as shown in Table 2.

**Table 2:** Clinical timeline.

TIME line (d)	Events recorded
01	Persistant sadness, irritability, anxiety, sleep disturbance, intrusive memories, avoidance behavior, and tearful.
	Underwent complete blood count, random blood sugar, liver function tests, renal function tests according to the protocols of our hospital and psychiatric evaluation was done.
02	Under care in medical unit, with anxiolytics and general support.
	Complete blood count repeated.
03	Hypervigilance, memory gaps, irrelevant talk, and headache.
	Computed tomography scan of brain.
04	Fever, confusion.
	Magnetic resonance imaging brain, lumber puncture performed, and cerebrospinal fluid sent for routine examination.
	Intravascular acyclovir started.
05	Altered mental status.
	Cerebrospinal fluid polymerase chain reaction done which revealed herpes simplex virus.
09	Flash backs, irrelevant talk but cooperative.
10	Vocal, improved, reassured, and discharged from hospital.
15	Mild sadness on follow-up, reassured and follow-up advised after 2 weeks.
30	Attentive, oriented to time, place and person, no complaints.

## 3. Discussion

HSV-1 encephalitis is the most common cause of sporadic viral encephalitis worldwide, with an incidence of 2 to 4 per million annually.^[6–8]^ It is a neurologic emergency with mortality >70% without treatment.^[9]^

While psychiatric mimicry of HSV encephalitis has been described,^[10]^ this case underscores the coincidence of DSM-5—defined acute stress disorder and HSV encephalitis, creating a diagnostic pitfall. The early psychiatric label delayed suspicion for infection, nearly leading to mismanagement.

CT is often normal in early HSV encephalitis, with sensitivity <30% in the first 72 hours.^[11]^ MRI, by contrast, is more sensitive (>90%), especially for temporal lobe involvement.^[12]^ In this case, a vigilant trainee’s recognition of subtle CT hypodensity prevented premature discharge, highlighting the importance of careful image review.

Clinicians should maintain a high index of suspicion when psychiatric symptoms present with sudden onset or atypical progression. Early neuroimaging and cerebrospinal fluid (CSF) analysis are essential for accurate and timely diagnosis, as they can uncover underlying viral encephalitis that initially mimics primary psychiatric disorders.

Stress is a well-established reactivation trigger, mediated by neuroendocrine–immune interactions. Catecholamines and glucocorticoids suppress innate immunity, impair interferon responses, and enhance viral replication.^[13–15]^ Acute psychological stress, such as bereavement, may therefore play a contributory role in reactivating latent HSV infections, as observed in this case.

Differential diagnosis included worsening ASD, psychogenic agitation, metabolic encephalopathy, and viral encephalitis. Only after progression and confirmatory CSF testing was HSV diagnosed.

The patient recovered fully after acyclovir, consistent with reports showing that early antiviral initiation improves prognosis dramatically.^[16,17]^ At a 1 month, no cognitive or psychiatric sequelae were detected.

## 4. Conclusion

Our case illustrates how psychiatric symptoms may mask early HSV encephalitis, delaying recognition of a life-threatening infection. Clinicians must remain vigilant when psychiatric conditions show atypical progression (e.g., fever, confusion, neurologic decline). Timely MRI and CSF studies are critical for early diagnosis.

The case also highlights the potential role of acute stress as a trigger for HSV reactivation, emphasizing the need for multidisciplinary collaboration across psychiatry, neurology, and radiology.

## Acknowledgments

We thank the patient for providing consent for this case report and the medical team for their dedication to patient care.

## Author contributions

**Conceptualization:** Riazullah.

**Data curation:** Riazullah, Aafeen Mujeeb, Muhammad Saqib, Inibehe Ime Okon.

**Formal analysis:** Muhammad Saqib.

**Investigation:** Inibehe Ime Okon.

**Methodology:** Muhammad Saqib, Inibehe Ime Okon.

**Project administration:** Riazullah.

**Resources:** Aafeen Mujeeb.

**Software:** Muhammad Saqib.

**Supervision:** Inibehe Ime Okon.

**Validation:** Riazullah, Aafeen Mujeeb, Inibehe Ime Okon.

**Visualization:** Riazullah.

**Writing – original draft:** Riazullah, Aafeen Mujeeb, Muhammad Saqib, Inibehe Ime Okon.

**Writing – review & editing:** Riazullah, Aafeen Mujeeb, Muhammad Saqib, Inibehe Ime Okon.
